# Editorial: Apoptosis, autophagy, and mitophagy dysfunction in Alzheimer's disease: Evolving emergence and mechanisms

**DOI:** 10.3389/fnmol.2022.1049914

**Published:** 2022-10-11

**Authors:** Rajkumar Singh Kalra, Ramesh Kandimalla, Binukumar BK

**Affiliations:** ^1^Immune Signal Unit, Okinawa Institute of Science and Technology Graduate University, Okinawa, Japan; ^2^Department of Biochemistry, Kakatiya Medical College, Warangal, India; ^3^Applied Biology, Council of Scientific and Industrial Research-Indian Institute of Chemical Technology, Secunderabad, India; ^4^CSIR Institute of Genomics and Integrative Biology, New Delhi, India

**Keywords:** apoptosis, autophagy, mitophagy, Alzheimer's disease, amyloid-β plaques, neuro-fibrillary tangles, neuroinflammation, neurodegeneration

Apoptosis and autophagy are intrinsic processes essentially required for cellular homeostasis (Song et al., 2017). Irreparable DNA damage or critical oxidative stress often triggers apoptosis to clear the damaged cell from the tissue (Delhalle et al., 2003). The distinct morphology of apoptosis includes cytoskeletal collapse, cytoplasmic condensation and fragmentation, membrane blebbing, nucleus pyknosis, chromatin condensation and fragmentation, and the formation of membrane-enveloped apoptotic bodies that are subsequently phagocytosed by macrophages and adjacent cells (Delhalle et al., 2003). Apoptosis plays a vital function in sustaining neural and brain health (Wu et al., 2015). Autophagy also promotes the health of the brain and nervous system by systematically eliminating damaged organelles and aging cytosolic proteins (Song et al., 2017). Autophagy progress through a series of events that includes the formation and elongation of double-membrane, vesicle maturation, and subsequent removal of targeted material by the lysosome (Wu et al., 2015; Dewanjee et al., 2021). Neurons, specialized post-mitotic cells, inherently rely on high mitochondria output to aptly fulfill their greater bioenergetics need, wherein the role of mitochondrial quality control mechanisms such as mitophagy is critical in sustaining neuronal health and function (Lou et al., 2020). Recent research in the field shows that the molecular processes of apoptosis, autophagy, and mitophagy are impaired in Alzheimer's disease (AD); however, the onset and progression of these mechanisms are elusive (Tran and Reddy, 2020). Moreover, declined physiological functions in aging patients were suggested to be a result of altered function of metabolic pathways (Kalra et al., 2021),

which are often seen intermingled with apoptosis, autophagy, and mitophagy (Tran and Reddy, 2020). Weighing the evolving importance of the impaired oxidative stress, mitochondria, and lysosomes pathways in neurodegenerative diseases (Shefa et al., 2019), a thematic focus of this Research Topic was outlined to gather new knowledge on the intricated apoptosis, autophagy, and mitophagy processes in the neurons/brain and their dysfunctions in AD by shedding light on their emerging associations, clinical significance, and translational importance.

In this line, the original research article(s) and reviews published in the present Research Topic elucidate/discuss new findings on apoptosis, autophagy, and mitophagy dysfunction in AD and shed light on their emerging clinical significance and therapeutic importance.

In an original report, Subui et al. investigated mitochondrial transport and distribution in tauopathy neurons (Figure 1). Given the fact that mitochondria play a critical role in neuronal homeostasis, where it supplies ATP and buffer Ca2+ at synaptic terminals, intricate neuron structural geometry causes a major hurdle in transporting mitochondria to synaptic ends. In this practice, mitochondria are delivered by kinesin motors at the axonal compartments, while retrograde transport is performed by cytoplasmic dynein. Understanding axonal mitochondrial transport and dispersal in tauopathy neurons is critical to evaluate their significance in AD progression. To this end, they observed a reduced anterograde mitochondria transport and its axonal abundance in P301L neurons; however, retrograde transport remained unchanged. Of note, while analyzing the involvement of motor proteins, a significant decline in the mitochondrial association of kinesin in P301L cells was observed. Furthermore, they suggested a possible surge in dynein activity to support retrograde flux in the P301L cells. Conclusively, the authors suggested that reduced kinesin-mediated transport with ongoing retrograde transport may result in reduced axonal mitochondria density in tauopathy neurons, potentially instigating synaptic deficits in AD and other tauopathies.

**Figure 1:**
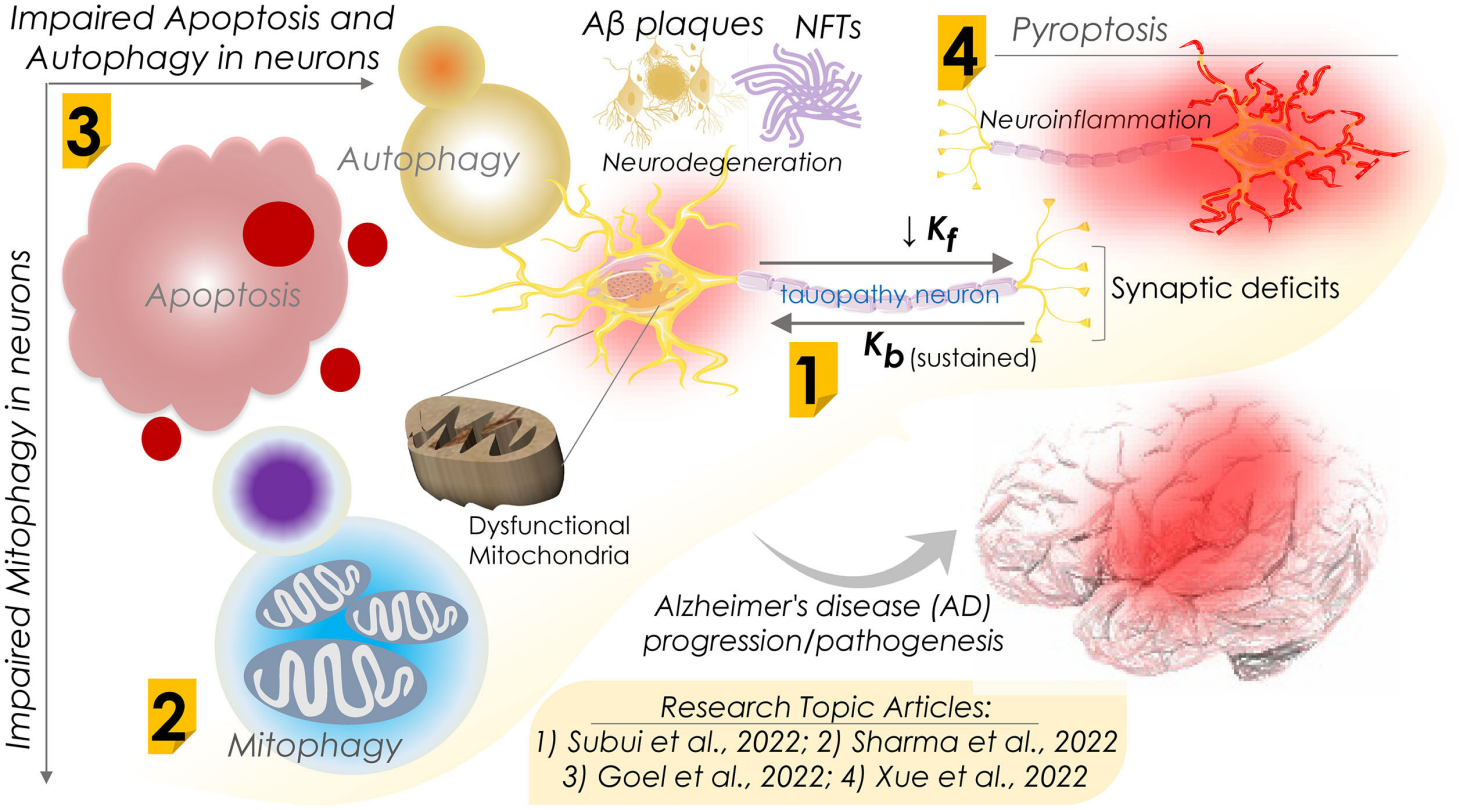
Schematic diagram showing dysfunctions of apoptosis, autophagy, mitophagy, and pyroptosis in Alzheimer's disease progression and their mechanisms. Research Topic articles are denoted by serial number within the related schematic section; k_f_ and k_b_ represent kinesin and dynein mediated flux, respectively.

Defective mitochondrial function is often seen in neurodegenerative diseases, including AD. Accumulating impaired and malfunctioning mitochondria is primarily believed to be an early sign of AD progression. Mitophagy, a naturally evolved selective mitochondrial autophagy, is a critical mitochondrial quality control process that sustains neural health and function by removing defective mitochondria. To shed light on the mitophagy function in AD, in a comprehensive review, Sharma et al. assessed the association of impaired mitochondria and dysregulated mitophagy with the onset of AD (Figure 1). Several proteins associated with mitophagy exhibited an altered expression in AD and were considered key therapeutic targets. Therefore, therapeutic regimes targeting these proteins and restoring mitophagy capabilities could be novel interventional strategies against AD. Furthermore, they summarized the emerging significance of mitophagy in the onset and development of AD and the mitochondrial quality control mechanisms. They also outlined the outcome of dysfunctional mitophagy in AD and underlined the pressing need for therapeutic approaches involving the modulation of mitophagy function in AD. However, the authors stressed the necessity of a more detailed investigation to delineate the role of mitophagy in the pathophysiology of AD.

In contrast to mitophagy, neuronal cell death mechanisms in AD are of significant therapeutic interest. Regulated cell death (RCD) is a well-ordered and orchestrated program that involves both genomic and proteomic components to regulate normal development and tissue homeostasis. Aberrant activation of RCD results in cell death by multiple mechanisms, including apoptosis, necroptosis, ferroptosis, pyroptosis, and autophagy-dependent cell death. In a review report, Goel et al. assessed the association of these mechanisms in neurons with the pathogenesis of AD and other neurodegenerative disorders (Figure 1). As the pathological hallmarks, AD diagnosis primarily checks for accumulating two key protein markers, amyloid β peptides and atypically phosphorylated tau protein. A pathological outcome of this is the formation of protein aggregates that include beta amyloid plaques and neuro-fibrillary tangles (NFTs). Beta amyloid plaques and NFTs-induced neuroinflammation and neurodegeneration have been studied extensively and are believed to initiate cognitive and behavioral deficits. Of note, the autopsy of AD patients' brains exhibits vast neuronal death as evidenced by cortical volume shrinkage, reduced gyri (to up 50%), and increased sulci sizes. However, the distinct mechanism(s) that triggers neuronal cell death in AD patients remains elusive owing to the limited availability of dying neurons. Authors in this review report attempted to evaluate the processes of RCD known to date, including the impaired response to diverse intra and extracellular stressors, types of dysregulated cell death, their interplay, and function in AD pathogenesis in humans and animal models of AD. Moreover, they assessed the correlation of both amyloid-β and Tau pathologies with neuronal loss in AD.

In this line, Xue et al., in a review report, further evaluated research progress on pyroptosis in AD (Figure 1). Assessing the complex pathogenic mechanism of AD, where neuroinflammation and neural cell death are key contributors, the authors reviewed the progressive neurodegeneration in AD and its clinical manifestations, including memory and visuospatial cognitive decline. Pyroptosis, i.e., an inflammatory programmed cell death, is primarily seen to be involved in neuron death in AD. Pyroptosis is performed by the gasdermins family proteins that form pores in the plasma membrane on receiving the upstream signals (NLRP3 and caspases) and trigger the inflammatory cascades marked by interleukin (IL)−1β and IL-18 release. Conclusively, the authors attempted to summarize recent findings highlighting the roles of neuron pyroptosis in AD and further discussed their molecular mechanisms in AD pathogenesis.

In summary, the present Research Topic gathers original research and comprehensive reviews highlighting the role of apoptosis, autophagy, and mitophagy dysfunctions in AD pathogenesis. It further comprises new knowledge on these processes's functional and pathophysiological emergence and their underlying mechanisms that could advance AD diagnosis and therapeutics in clinics.

## Author contributions

All authors listed have made a substantial, direct, and intellectual contribution to the work and approved it for publication.

## Conflict of interest

The authors declare that the research was conducted in the absence of any commercial or financial relationships that could be construed as a potential conflict of interest.

## Publisher's note

All claims expressed in this article are solely those of the authors and do not necessarily represent those of their affiliated organizations, or those of the publisher, the editors and the reviewers. Any product that may be evaluated in this article, or claim that may be made by its manufacturer, is not guaranteed or endorsed by the publisher.
